# A Study on the Contributions of Sonication to the Identification of Bacteria Associated with Intubation Cannula Biofilm and the Risk of Ventilator-Associated Pneumonia

**DOI:** 10.3390/medicina59061058

**Published:** 2023-05-31

**Authors:** Ioana Roxana Codru, Mihai Sava, Bogdan Ioan Vintilă, Alina Simona Bereanu, Victoria Bîrluțiu

**Affiliations:** 1 Faculty of Medicine, Lucian Blaga University, 2A, Lucian Blaga Str., 550169 Sibiu, Romania; mihai.sava@ulbsibiu.ro (M.S.); alina.bereanu@ulbsibiu.ro (A.S.B.); victoria.birlutiu@ulbsibiu.ro (V.B.); 2 County Clinical Emergency Hospital, 2–4, Corneliu Coposu Bld., 550245 Sibiu, Romania

**Keywords:** ventilator-associated pneumonia, biofilm, sonication

## Abstract

Ventilator-associated pneumonia is one of the most severe complications of critically ill patients that need mechanical respiratory support, as it poses a significant risk of prolonging hospitalization, disability, and even death. This is why physicians worldwide target newer methods for prevention, early diagnosis, and early target treatment for this condition. There are few methods for a quick etiological diagnosis of pneumonia, especially point of care, and most are only readily available in some intensive care units. This is why a new, simple, and cheap method is needed for determining the bacteria that might be infectious in a particular patient. The manner in question is sonication. Method: In this prospective, observational, single-center study, endotracheal cannula specimens will be collected from at least 100 patients in our intensive care unit. This specimen will be submitted to a specific sonication protocol for bacteria to dislodge the biofilm inside the cannula. The resulting liquid will be seeded on growth media, and then a comparison will be made between the germs in the biofilm and the ones in the tracheal secretion of the patient. The primary purpose is to determine the bacteria before the appearance of a manifest infection.

## 1. Introduction

The most prevalent infection in the intensive care unit (ICU) is the hospital–acquired pneumonia (HAP) [1,2]. This group of conditions encompasses two different entities: ventilator-associated pneumonia (VAP) and high-severity pneumonia developed during hospitalization. VAP is a type of pneumonia that develops in mechanically ventilated patients. The reported incidence of VAP is extensive, ranging from 1.9–3.8 per 1000 days of mechanical ventilation in the USA to more than 18 per 1000 days in Europe [1]. The incidence of this type of pneumonia in Romania is probably even higher, but retrospective or prospective multicentric studies have not quantified its prevalence [2].

VAP occurs after 48 h of mechanical ventilation. The onset relative to hospital admission discriminates early pneumonia (under five days) from late pneumonia (over five days) [3,4].

VAP diagnosis is made on clinical, paraclinical, and radiological criteria. For the radiological criterion to be met, two successive chest radiographs showing new or progressive lung infiltrates or a single chest radiograph without a medical history of underlying heart or lung disease are required. It should be accompanied by at least one of the following: new onset fever (without any other cause) or changes in the leukocytes number (≤4000 mm^3^ or ≥12,000 mm^3^) and at least two of the following signs: purulent sputum, (cough, dyspnoea—for HAP), declining oxygenation, increased oxygen requirements(4).

The most common causes of VAP are bacteria, but viruses are increasingly recognized. Fungal pathogens are uncommon except for immunocompromised patients [5] and patients treated with wide-spectrum or long-term antibiotic treatments. The frequency of different pathogens can vary according to geographic region and according to hospital and hospital clinical activity. The bacteria most frequently isolated from patients with VAP are aerobic gram-negative bacteria (Klebsiella pneumoniae, Acinetobacter species, Pseudomonas aeruginosa, Enterobacter spp, Stenotrophomonas maltophilia, Serratia marcescens) in more than 60% of the cases [6,7,8] and gram-positive cocci (Staphylococcus aureus, particularly MRSA). The rates of polymicrobial infections vary even more and are usually associated with aspiration, and the incidence is higher in adults with acute respiratory distress syndrome (ARDS) [9]. The viral etiology of hospital-acquired pneumonia appears most commonly seasonal, including influenza, parainfluenza, adenovirus, and respiratory syncytial virus [9]. SARS-CoV has become one of the most prevalent viruses to be isolated in hospitalized patients in the last four years.

The complications associated with ventilation-associated pneumonia are multiple. Prolonged antibiotic therapy and mechanical ventilation increase the risk of serial colonization and reinfection with pathogens: recurrent pneumonia or Clostridium difficile colitis [5]. A possible risk is a cardiac decompensation triggered by the combination of hypoxemia and increased metabolic demands due to infection. It can manifest by acute ischemia, exacerbation of heart failure, and new onset arrhythmias [10].

A pooled analysis of randomized studies shows that attributable mortality from ventilator-associated pneumonia is estimated at 10% [10]. About one-third of HAP develops in ICU, with VAP accounting for 90% of cases. VAP occurs in 9–40% of intubated patients, representing the most frequent ICU-acquired infection [11]. The 28-day mortality rate for hospital-acquired pneumonia is around 30% among patients admitted to the ICU [12].

Along with the increased morbidity and mortality associated with VAP, this kind of healthcare-associated infection is of concern as it poses a substantial economic burden. The Society of Healthcare Epidemiology of America (SHEA) conducted a matched cohort study of the Premier database. It evaluated the impact of VAP on the length of stay (LOS) in the hospital and ICU, duration of mechanical ventilation, and hospital costs. Regarding expenses, SHEA demonstrated an increase in hospitalization costs by almost 40% [13].

Currently, few measures try to prevent ventilator-associated pneumonia, and even fewer cheap and accessible means of early diagnosis of this condition. Early diagnosis is essential as it prompts targeted antibiotic therapy and a faster resolution of pneumonia. The microbiologic tests (phenotypic, molecular, and rapid tests) have a series of advantages as an etiological diagnostic method for infection. Phenotypic tests are time-consuming, have limited ability to differentiate closely related species, and may not detect non-viable or slowly growing bacteria. With high sensitivity and specificity, molecular tests necessitate specialized equipment and expertise. Rapid tests, on the other hand, though quick and reliable, may have a lower sensitivity compared to molecular tests and may sometimes need validation or confirmation using other methods. The main disadvantage of the previously described methods is the lack of availability and the increased costs.

The purpose of using sonication as a detection method is to develop a cheap, sensitive, and specific method for microorganism detection from medical biofilms, including the ones formed on the intubation cannulas.

As there are many bio-film-related infections, from catheter-associated urinary tract infections to central line-associated bloodstream infections [14,15,16], one can only imagine that the intubation cannula can provide a perfect surface for bacteria to adhere to.

Sonication applies sound energy to agitate particles or discontinuous fibers in a solution or dislodge cells from certain surfaces [17]. The acoustic energy or the sound wave involves the conversion of an electrical signal into a physical vibration with a specific frequency and amplitude directed toward a substance [18,19].

Sonication was used mainly in domains only tangent to the medical field: pharmaceutical and cosmetics, and other industries, such as food, water, pesticides, ink, paint, coating, nanocomposite, metalworking, wood product, wood treatment, and many others. It is helpful with the production of nanoparticles such as nanoemulsions [20], nanocrystals, liposomes, and wax emulsions, as well as for water purification, degassing, extraction of seaweed polysaccharides [17] and plant oil, extraction of anthocyanins and antioxidants [21], production of biofuels, crude oil desulphurization, cell disruption, polymer and epoxy processing, adhesive thinning, and a lot of other processes.

In the last decade, sonication has become more and more influential in the biomedical field as the method used to determine the bacteria associated with different kinds of biofilms.

To use sonication for microorganism dislodgement from different surfaces, a low sonication condition is needed to obtain viable bacteria. It represents a low-intensity technique, which is not destructive and consists of a series of sound waves with certain amplitude, frequency, distance, time, and temperature [17,22].

The immediate effect of sound is the modification of the permeability of the cellular plasma membrane by applying the acoustic cavitation of microbubbles to enhance delivery. This specific phenomenon is called sonoporation [23]. Depending on the expected result, sonoporation can have beneficial or undesirable effects. Due to the possibility of the appearance of cavitation in aqueous solutions, the cell wall of the microorganisms is at risk of destruction [17,22,23]. Due to the collapse of the bubbles, a high shear force is generated in the environment that breaks the cell wall and membranes [24]. Thus, if the purpose of sonication is to dislodge the bacteria from the surfaces and to keep them viable, the variables of the sound waves must be adjusted so that the plasmatic cell membrane remains intact. The ultrasound waves can then have two types of effects based on the result on the microorganism: reversible (or repairable), when the pores induced in the cell membrane can reseal, leading to cell survival, and irreversible (or lethal), when the cell dies because of the cellular lysis [23,25].

Studies performed in vitro demonstrated that different bacterial species have additional resistance to sound waves. If the sonication protocol is also associated with increases in the temperature of the environment, the destruction of the bacteria is obtained. Gram-positive bacteria were generally more resistant to the effect of ultrasounds than gram-negative ones [26,27]. The layer of proteoglycans in the gram-positive cellular wall is usually much thicker than that in the gram-negative microorganisms. Lipopolysaccharides are the factors that significantly contribute to the structural integrity and protect the membrane of the latter category. In this case, the set target for the sound waves should be the inner layer of the membrane, the one consisting of lipopolysaccharides [28]. On the other hand, spores, for instance, are very resistant to sonication. There has been a report by Pitt and Ross [29] affirming that the cell wall of the spore can even grow under the influence of low sonication (≤2 W/cm^2^). It is the consequence of pore formation in the cell wall, which facilitates the transport of nutrients and small particles (water, carbon dioxide, peptides, and amino acids) in the solution, and of the inability of ultrasound to remove cells from the surfaces altogether.

It is well known that many different microorganisms can adhere to surfaces and form biofilms, but the adherence strength is different. Sonication can dislodge any biofilm, but not all bacteria can be easily cultivated on media because some necessitate special growth media and environments, and others are killed before inoculation. This being said the precipitation of the sonication fluid is more likely to contain gram-positive bacteria, mainly cocci and gram-negative ones. Particular types of microorganisms, such as mycobacteria or anaerobes, will not be isolated.

## 2. Methods

The present study is a prospective, observational, single-center study. It aims to isolate the bacteria in the biofilms of the tracheal cannulas of mechanically ventilated patients. The purpose of bacterial isolation is not only to compare with the bacterial load from tracheobronchial secretion but also to determine the optimal replacement time of the endotracheal cannulas in patients with prolonged mechanical ventilation.

It is estimated that more than 100 mechanically ventilated patients will be enrolled in the study over 12 to 18 months. The study will be located in the intensive care unit of Sibiu County Clinical Emergency Hospital, Romania. This ICU is a level I intensive care as it can assist a large variety of pathology: medical and surgical (general, thoracic and vascular surgery, neurosurgery, ENT, urology, trauma, and burns). It is organized on two floors with 12 single-bed rooms on each floor and four double-bed rooms for post-anesthesia care/intermediary care. 

This study will not affect the patient’s treatment plan and will be performed according to the attending physician’s indications. The endotracheal cannulas will be replaced if the patient’s clinical assessment allows this maneuver without endangering the well-being of the ill.

### 2.1. Aims of the Study

Detection of the bacteria in the biofilm formed inside the endotracheal cannulae.

Detection of the mean period during which the biofilm organizes in the lumen of the cannulae.

Determination of the optimal time to replace the intubation cannulae in patients that need mechanical ventilation for more than 48 h so that the contamination of the lower respiratory airways by dislodging the biofilm following suction maneuvres is prevented. 

Comparison of the microorganisms from the patient’s respiratory secretions with the bacterial load in the biofilm to initiate an early targeted antibiotherapy. Furthermore, it should be determined whether a clean cannula with a sterile biofilm can prevent the onset of VAP. 

Development of new methods to prevent VAP.

Final aim: decreasing the morbidity and mortality of critically ill patients by preventing or early and targeted treating VAP, reducing the LOS in the ICU and in the hospital with a secondary lowering of costs associated with hospitalization.

### 2.2. Population Description

Patients admitted to our ICU, aged between 18 and 100 years old, are mechanically ventilated for more than 48 h. Among the causes of respiratory failure are pulmonary infections (bacterial, viral, or fungal pneumonia, bronchopneumonia, COPD exacerbations due to respiratory tract infections), aggravated lung diseases (severe asthma attack, status asthmaticus), or extrapulmonary factors (polytrauma after traffic accidents, falls, neurological or neurosurgical patients with deteriorated consciousness and alterations of the airway reflexes).

### 2.3. Inclusion Criteria

Age between 18 and 100 years old.

Romanian citizenship.

Patients or legal representatives are informed and consent in writing to be part of the study.

Patients that require more than 48 h of mechanical respiratory support.

Exclusion criteria:

Under-aged patients.

Patients who do not consent or the consent could not be obtained.

The non-compliant collection of biological samples in patients.

Clinically unstable patients, so the samples could not be collected.

Less than 48 h of mechanical ventilation.

Non-Romanian nationality.

Eligible patients for the proposed research topic will be divided into two groups according to the cause of respiratory failure:

Group 1—respiratory failure due to pulmonary infection—confirmed bacterial pneumonia, bacterial bronchopneumonia, or with a high degree of clinical and biological suspicion (purulent tracheal secretions, paraclinical or imaging investigations highly suggestive of respiratory infection).

Group 2—respiratory failure secondary to non-infectious pulmonary conditions (severe asthma attack, pulmonary fibrosis) or extrapulmonary causes that need mechanical ventilation (polytrauma secondary to traffic accidents, falls, neurological or neurosurgical patients, patients with deteriorated consciousness that need airway protection).

### 2.4. Data Collection

After admission to the ICU, a quick but rigorous clinical exam is performed to stabilize or correct the issues that can immediately endanger the patient’s life, according to the ABC rule (airway, breathing, circulation). If the patient is already mechanically ventilated, the patency of the tube is checked, and specific ventilatory parameters will be set to ensure adequate ventilation and respiration of the critically ill. If the patient breathes spontaneously but needs respiratory support, orotracheal intubation will be performed, and mechanical ventilation will be initiated. The same measures are taken in the case of the patient that is already in the ICU but has deteriorated. 

Data:

Age, gender, BMI.

Length of stay in hospital.

Length of stay in ICU.

Number of days of stay in ICU before intubation.

Outcome.

Mortality at 28 days.

Diagnostic criteria for ICU admission.

Cause of the respiratory failure.

Associated conditions.

Sickness severity scores (SOFA, APACHE II).

Pneumonia severity scores (PSI/Pneumonia severity index; SMART-COP Score; CURB-65).

Blood work (including inflammatory panel and blood gas analysis).

Monitoring curves (blood pressure, heart rate, oxygenation, ventilation, diuresis, GFR, creatinine clearance).

Antibiotherapy.

Newly onset organ dysfunction/worsening of a preexisting organ dysfunction/organ failure.

### 2.5. Microbiological Specimens—The Timing of the Sampling, Specimen Manipulation, Sonication Protocol

T_0_: tracheal aspirate collection in the first 2 h after the patient’s admission to the intensive care unit or after tracheal intubation and initiation of the invasive respiratory support.

Collection of a second tracheal aspirate 48–72 h after T_0_.

Replacement of the endotracheal cannula at 48–72 h from T_0_ and collection of a cannula specimen. The specimen will be sonicated according to an established protocol for bacterial sonication, and the sonication fluid will be inoculated onto bacterial culture media.

Collection of a third tracheal aspirate at 168–192 h from T_0_ if the patient requires prolonged mechanical ventilation or required reintubation less than 24 h from the time of extubation.

Change of the endotracheal cannula at 168–192 h from T_0_ and the collection of the cannula specimen, sonication, and fluid seeding onto growth media.

Sonication protocol: Orotracheal intubation cannula specimens will be sonicated for 30 min using an ultrasonic bath (BactoSonic14.2, Bandelin GmbH, Berlin, Germany) at a frequency of 42 kHz with a power of 0.22 W/cm^2^. The resulting sonication liquid is then homogenized, and 5–10 mL is centrifuged for 5 min at 2500 rpm. The resulting precipitate will be inoculated onto culture media and incubated at 37 degrees to inspect them for bacterial growth.

### 2.6. Ethics and Personal Data Protection

This study has obtained ethics approvals from the ethical committee of the County Clinical Emergency Hospital of Sibiu and the Lucian Blaga University of Sibiu. To be enrolled in the study, the patient or the legal representative must sign an informed consent. The consent can be withdrawn at any point without impacting the patient’s treatment and care.

According to Romanian law, the information collected for this study will be confidential, and patient data will not be published. Access to personal data is provided only to the research team involved in the study. However, the control committees will be granted access to the initial patients’ data to verify compliance with the study procedures. However, they will not be allowed to make the data public.

## 3. Discussions

Since critically ill patient represents an enormous challenge no matter the admission diagnosis, the primary aim of the healthcare system is to decrease the mortality and morbidity of the patients and shorten the length of stay in intensive care units. By achieving these targets, the total costs of care will be significantly lower. More than other complications, VAP can extend the length of ICU stay and pose the patient with a significant risk of death. Many studies focus on preventing VAP, early diagnosis, and targeted treatment. 

At this point, though, there is no prophylactic measure that is 100% efficient, and the rapid diagnostic methods (PCR methods for bacterial isolation) are not readily available in all intensive care units. This is the main reason for using sonication as a new prevention and early diagnosis method. Sonication is cheap, and there is no need for highly trained personnel or expensive reactants. This kind of method was used in clinical practice sparingly. Dentistry is the main branch that uses sonication to determine the bacteria in the biofilms of dental prostheses. In the last years, though, steps in using sonication were made in orthopedy, when this method was used to isolate bacteria from infected hip or knee prostheses. Because the traditional methods could not isolate any microorganism, the biofilm was dislodged with the help of the ultrasounds, and a rare bacteria were isolated: Ralstonia pickettii. The patient with a hip infection with R. picketii could be treated with targeted antibiotics after the bacteria responsible for the infection was finally isolated [30].

As this method progressed in orthopedy, more doctors used sonication to isolate bacteria on the infected knee and hip prostheses. In a recent study, the authors isolated many strains of bacteria. There were found gram-positive bacteria and also gram-negative bacteria with different kinds of resistance to antibiotics. Though the clinical signs and symptoms suggest prosthetic infection, no microorganism could be isolated through classic methods. Using sonication on infected hip or knee prostheses extracted from patients, the authors isolated numerous strains of microorganisms: Coagulase-negative staphylococci (CoNS) species—epidermidis, lentus, xylosus, epidermidis, hominis, hemolyticus; Enterococcus faecalis, Escherichia coli, Klebsiella spp, Proteus mirabilis, Pseudomonas spp and even Acinetobacter spp. Along with sonication, other tissue samples were collected in the intraoperative period: one was used for the histopathological examination, the prosthetic membrane, and the others were sent for bacterial cultures in the microbiology laboratory [31].

For orthopedic patients, to use sonication on the prosthesis, there is a need for genuinely invasive procedures. On the other hand, the invasivity is very low in dentistry, at least in the case of totally mobile dental prostheses. In the intensive care unit, we are between orthopedy and dentistry to get the specimen for sonication, as a piece of the intubation cannula is needed.

As with any diagnostic method, sonication has its limitations. First, the sonication fluid is needed. If the patient is not stable enough (there are difficulties in maintaining proper oxygenation or the cardiovascular system needs increasing vasoactive or positive inotrope support), the tracheal cannula cannot be changed in the proposed timeline without endangering the ill. In this scenario, the cannula will stay on the spot, and the patient will be excluded from the study. Even though it enhances detection sensitivity compared to traditional culture methods, it may cause cell damage and loss of viability, and furthermore, cannot differentiate between viable or nonviable microorganisms.

Another area for improvement regarding this study is the impossibility of isolating certain species of bacteria. Bacteria that require special growth media can be included in this category: atypical bacteria, mycobacteria, and anaerobic bacteria. It has already been described in the medical literature that mycobacteria have the ability to adhere to surfaces and form biofilms [32]. The ultrastructure of the mycobacterial biofilms has been studied through different kinds of methods: Confocal laser scanning microscopy (CLSM) combined with fluorescent dyes, Nile Red, or LIVE/DEAD BacLight. This type of coloration is used to differentiate between the mycobacterial species by analyzing the phenotypic characteristic of biofilms (growth rate, live and dead bacteria, autofluorescence) [33]. Most anaerobes adhere strongly to surfaces, as G. Donelli and Co concluded after studying biliary stents biofilms. Furthermore, they discovered that a plurimicrobial biofilm is even more adherent than the monomicrobial one, so the energy of the sound wave used for the dislodgement should be higher. After the dislodgement, the bacteria were grown on specific media brain heart infusion (BHI) broths and then colored with Hucker crystal violet. The colored and then dried plates were examined by using microplate photometers. Even further, field emission scanning electron microscopy (FESEM) or confocal laser scanning microscopy (CLSM) was used [34]. As there are methods described in the literature to evaluate biofilm formation and composition, most of them are expensive and difficult to use. They cannot be used as a bedside method and are not readily available for all intensive care units or hospitals worldwide.

The proposed number of patients to be included in the study is at least 100. This figure might not be achieved if the number of intubated and ventilated patients in our ICU is lower than the number proposed or if other exclusion criteria are met on the way (withdrawn consent, less the 48 h of mechanical ventilation).

The possible upgrades of the current study can also be added if it contained a sub-study about antibiotic activity on the biofilm of the intubation cannula as compared to the antibiotic activity on the bacteria isolated from the tracheobronchial secretions. Furthermore, it is essential to know the difference between the minimal inhibitory concentration (MIC) of the two types of bacteria. The literature has already published that the antimicrobial concentrations needed to sterilize biofilms are more significant than those needed to eradicate the same bacteria in a planktonic state [35]. It is well known that bacteria contained in biofilms have overwhelming survival capabilities to multiple classes of usual antimicrobials [36,37], even when it is not considered the microorganism’s MDR status. Knowing this, the patients can be treated accordingly, using the appropriate doses of antibiotics in this subtype of population: the infected critically ill patient admitted to the intensive care unit.

## Data Availability

All data generated and analyzed during this study are included in this published article.

## Abbreviations

*ICU* (Intensive Care Unit), *HAP* (Healthcare Associated Pneumonia), *VAP* (Ventilator-associated Pneumonia), *MRSA* (Methicillin-resistant Staphylococcus aureus), *SHEA* (The Society of Healthcare Epidemiology of America), *LOS* (Length of stay), *ENT* (Ear, Nose, Throat), *BMI* (Body Mass Index), *SOFA* (Sequential Organ Failure Assessment), *APACHE* (Acute Physiology and Chronic Health Evaluation), *SMART-COP* (Systolic blood pressure, Multilobar infiltrates, Albumin, Respiratory rate, Tachycardia, Confusion, Oxygen, pH), *CURB-65* (Confusion, Urea, Blood pressure, age ≥ 65 years), *PSI* (Pneumonia Severity Index), *GFR* (Glomerular filtration rate), *PCR* (Polymerase chain reaction), *CoNS* (Coagulase-negative staphylococci), *MIC* (minimal inhibitory concentration), *CLSM* (Confocal laser scanning microscopy), *FESEM* (field emission scanning electron microscopy), *BHI* (Brain Heart Infusion).
